# Nitric oxide debilitates the neuropathogenic schistosome *Trichobilharzia regenti* in mice, partly by inhibiting its vital peptidases

**DOI:** 10.1186/s13071-020-04279-9

**Published:** 2020-08-20

**Authors:** Tomáš Macháček, Barbora Šmídová, Jan Pankrác, Martin Majer, Jana Bulantová, Petr Horák

**Affiliations:** 1 Department of Parasitology, Faculty of Science, Charles University, Prague, Czechia; 2 Present Address: Center for Advanced Preclinical Imaging, First Faculty of Medicine, Charles University, Prague, Czechia

**Keywords:** Nitric oxide, Nitric oxide synthase, 3-Nitrotyrosine, Peroxynitrite, Cathepsin B, Schistosomatidae, *Trichobilharzia*

## Abstract

**Background:**

Avian schistosomes, the causative agents of human cercarial dermatitis (or swimmer’s itch), die in mammals but the mechanisms responsible for parasite elimination are unknown. Here we examined the role of reactive nitrogen species, nitric oxide (NO) and peroxynitrite, in the immune response of mice experimentally infected with *Trichobilharzia regenti*, a model species of avian schistosomes remarkable for its neuropathogenicity.

**Methods:**

Inducible NO synthase (iNOS) was localized by immunohistochemistry in the skin and the spinal cord of mice infected by *T. regenti*. The impact of iNOS inhibition by aminoguanidine on parasite burden and growth was then evaluated *in vivo*. The vulnerability of *T. regenti* schistosomula to NO and peroxynitrite was assessed *in vitro* by viability assays and electron microscopy. Additionally, the effect of NO on the activity of *T. regenti* peptidases was tested using a fluorogenic substrate.

**Results:**

iNOS was detected around the parasites in the epidermis 8 h post-infection and also in the spinal cord 3 days post-infection (dpi). Inhibition of iNOS resulted in slower parasite growth 3 dpi, but the opposite effect was observed 7 dpi. At the latter time point, moderately increased parasite burden was also noticed in the spinal cord. *In vitro*, NO did not impair the parasites, but inhibited the activity of *T. regenti* cathepsins B1.1 and B2, the peptidases essential for parasite migration and digestion. Peroxynitrite severely damaged the surface tegument of the parasites and decreased their viability *in vitro*, but rather did not participate in parasite clearance *in vivo*.

**Conclusions:**

Reactive nitrogen species, specifically NO, do not directly kill *T. regenti* in mice. NO promotes the parasite growth soon after penetration (3 dpi), but prevents it later (7 dpi) when also suspends the parasite migration in the CNS. NO-related disruption of the parasite proteolytic machinery is partly responsible for this effect. 
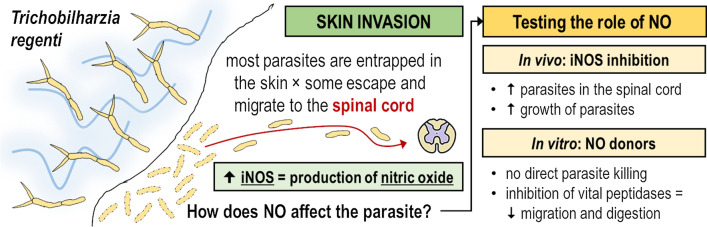

## Background

Reactive nitrogen species (RNS), such as nitric oxide (NO) or peroxynitrite (ONOO^−^), are essential antimicrobial molecules of the innate immunity [1, 2]. Upon stimulus, inducible NO synthase (iNOS) is activated and produces NO. It can bind to thiol groups of cysteines thus diminishing the function and stability of the affected proteins [3]. Furthermore, NO can couple with superoxide giving rise to ONOO^−^ which is a strong oxidizing and nitrating compound [4]. Formation of 3-nitrotyrosine (3-NT) is a general molecular marker of the nitrosative stress associated with RNS actions [5]. Besides amino acids, RNS also react with nucleic acids, unsaturated lipids or transition metal cations in Fe-S or Zn-S clusters, which impairs their physiological roles [6]. By targeting many diverse biomolecules, cytotoxic effects of RNS contribute to the control of both prokaryotic and eukaryotic pathogens [1, 2].

RNS-mediated host immune response is indispensable in fighting many parasitic agents. Apart from the well-known antimicrobial effects on parasitic protists, e.g. *Leishmania*, *Toxoplasma*, *Giardia* or *Trypanosoma cruzi* [7–10], RNS also participate in the control of infections caused by multicellular helminths, like schistosomes (blood flukes). One of the human species with major importance, *Schistosoma mansoni*, is prone to be damaged by NO which disrupts mitochondrial ultrastructure and causes lethal inhibition of aerobic energy metabolism [11–14]. Accordingly, only the life stages relying mostly on the mitochondrial respiration (skin and post-lung schistosomula) are affected by NO *in vitro* [11, 13, 15, 16]. The protective effect of NO, leading to lower parasite burden (i.e. number of parasites), was also demonstrated in control mice in comparison to those with pharmacological inhibition or gene knockout of iNOS [17, 18]. In rats, non-permissive hosts of *Schistosoma japonicum*, the high innate expression of iNOS was recently shown to block parasite growth, development of reproductive organs and egg formation, identifying NO as a key factor of the inherent resistance [14]. NO was also proposed to be co-responsible for host protection in mice vaccinated by *S. japonicum* calpain [19]. Considering the antiparasitic capacity of NO, novel antischistosomal therapeutics combining the traditional drug praziquantel with NO-releasing compounds were developed [20].

Apart from schistosomes, the important role of NO was suggested also in the control of other helminths such as *Trichinella spiralis* [21–23], *Brugia malayi* [24, 25], *Strongyloides venezuelensis* [26, 27] and *Taenia crassiceps* [28]. However, particular effector mechanisms of NO/RNS antiparasitic actions remain rather unexplored.

The avian schistosome *Trichobilharzia regenti*, used in this study, is closely related to the human blood fluke species. It infects anatid birds as definitive hosts but also penetrates the skin of mammals [29]. In humans, cercarial dermatitis (or swimmer’s itch) represents a neglected allergic condition which develops after repeated exposure to avian schistosomes [30, 31]. Being regarded as an emerging disease, cercarial dermatitis has substantial health and economic consequences, especially if outbreaks afflict recreational bathing areas [32–35].

The host immune response in the skin, the entrance gate of *T. regenti*, is crucial for halting the invasion. Cysteine peptidases, such as histolytic *T. regenti* cathepsin B2 (TrCB2), facilitate penetration of the skin [36]. Within 24 hours post-infection (hpi), neutrophils, eosinophils and macrophages are recruited and entrap the parasites [37]. In mice, this reaction eliminates 90% of the newly transformed schistosomula [37, 38]. The early phase of the infection is associated with a mixed Type 1/Type 2 immune response which peaks 7 days post-infection (dpi) [38, 39].

Interestingly, 10% of schistosomula manage to escape the initial immune response in the skin and move along the peripheral nerves towards the spinal cord [40]. They migrate mostly through the white matter and injure axons by feeding on neuronal myelin sheaths [41, 42]. The intestinal cysteine peptidases of *T. regenti*, such as cathepsin B1.1 (TrCB1.1), degrade myelin basic protein (MBP), a prevailing constituent of parasite diet [43–45]. The highest parasite burden in the CNS is observed around 7 dpi when schistosomula are localized mostly in the thoracic and cervical spinal cord [40, 41]. Neutrophils, macrophages and microglia accumulate around the schistosomula and presumably participate in their killing. *in vitro*, microglia and astrocytes were shown to produce NO after exposure to *T. regenti* antigens [46]. The neuroinflammation peaks 14 dpi and no living schistosomula are detected in the spinal cord 21 dpi [37, 41]. Taken together, mammals can effectively control the infection both in the skin and the spinal cord, but particular effector immune mechanisms responsible for *T. regenti* elimination are not fully understood.

Here we examined the role of NO in the immune response of mice infected with *T. regenti*. First, we assessed systemic NO production by analysis of serum nitrites/nitrates and localized iNOS and 3-NT in the infected skin and spinal cord. Then we evaluated the effect of iNOS inhibition on the parasite burden and tissue pathology. To complete our *in vivo* observations, we tested the effect of NO and ONOO^−^ on viability and ultrastructure of *T. regenti* schistosomula and on the activity of their vital cysteine peptidases, TrCB1.1 and TrCB2, *in vitro*. Our results suggest the antiparasitic role of NO based on the continuous debilitation of the parasites rather than on acute cytotoxicity.

## Methods

### Parasites

The life-cycle of *Trichobilharzia regenti* is maintained at the Department of Parasitology, Charles University (Prague, Czechia) using domestic ducks (*Anas platyrhynchos* f. *domestica*) and freshwater lymnaeid snails *Radix lagotis* as definitive and intermediate hosts, respectively [29]. The snails were illuminated for 1 h to trigger the shedding of cercariae, which were then collected, counted and used for infection of mice or *in vitro* transformation to schistosomula.

### Infection of mice and collection of tissue samples

Two ways of infection were used to get samples of infected mouse pinnae or spinal cords for further immunohistochemical analyses.

As for the pinnae, mice were subtly anaesthetized using ketamine (100 mg/kg) and xylazine (10 mg/kg) and their pinnae were immersed into a microtube with 500 cercariae in dechlorinated tap water for 30 min. At given time points (8, 24 and 48 hpi), mice were sacrificed by cervical dislocation, the infected pinnae were cut off and fixed in 4% (w/v) freshly depolymerized paraformaldehyde (PFA) in 0.1 M phosphate-buffered saline (PBS; pH 7.4) overnight at 4 °C.

To obtain the spinal cords, mice were infected by exposure to 2000 cercariae in a 50 ml water bath for 1 h. At the desired time points (3, 7, 14 and 21 dpi), mice were anaesthetized by inhalation of isoflurane, transcardially bled out and perfused by heparinized (10 IU/ml) PBS and 4% PFA. The spinal cord was carefully extracted and post-fixed in 4% PFA overnight at 4 °C.

In both cases, 8-week-old females of C57BL/6JOlaHsd mice (Envigo, Venray, Netherlands) were infected and uninfected individuals of the same age were used as a control group (referred to as “0 hpi” or “0 dpi”).

### Measurement of serum nitrites/nitrates

The blood was collected prior to the perfusion right after cutting the right atrium. The undiluted serum samples obtained after centrifugation of clotted blood were deproteinized by filtration through the Amicon Ultra-0.5 Centrifugal Filter Unit, 10 kDa (Merck Millipore, Burlington, USA). The concentration of nitrites/nitrates, degradation products of NO, was measured by Nitrate/Nitrite Colorimetric Assay Kit (Cayman Chemicals, Ann Arbor, USA) according to the manufacturer’s instructions. Briefly, the samples were treated by nitrate reductase for 3 h and then the Griess reagent was added. After 10 min, the absorbance was read at 550 nm. The analyte concentration was calculated according to the standard curve.

### Immunohistochemistry

After fixation, the pinnae and spinal cords were washed with PBS, gradually saturated by 10, 20 and 30% (w/v) sucrose in PBS and embedded in Tissue Freezing Medium (Leica Biosystems, Nussloch, Germany). Ten-micrometre sections were prepared (Leica Biosystems CM3050S cryostat) and used for immunohistochemical detection of iNOS and 3-NT. After rinsing with PBS, the sections were treated by 0.1% (v/v) Triton X-100 and 1% (w/v) bovine serum albumin (BSA) in PBS for 60 min. Antigen retrieval was performed by 1% sucrose in PBS treatment for 24 h at 4 °C [47]. After washing, the sections were incubated with primary antibodies (rabbit anti-mouse iNOS, polyclonal, 1:500, Thermo Fisher Scientific, Waltham, USA; mouse anti-3-NT, clone 39B6, 1:200, Abcam, Cambridge, UK) diluted in 0.1% Triton X-100 and 1% BSA in PBS overnight at 4 °C. Corresponding fluorescently labelled secondary antibodies were added for 1 h (goat anti-rabbit Alexa Fluor 594, donkey anti-mouse Alexa Fluor 488; both 1:1000, Thermo Fisher Scientific). Finally, the sections were washed with PBS, mounted in VectaShield with DAPI (Vectors Labs, Burlingame, USA) and observed by a fluorescence microscope (BX51, Olympus, Tokyo, Japan). Quick Photo Micro 3.0 and GNU Image Manipulation Program v.2.8. were used to acquire and process the images.

### Aminoguanidine treatment

Aminoguanidine hydrochloride (AG; Sigma-Aldrich, St. Louis, USA), the inhibitor of iNOS, was intraperitoneally administered to mice (60 mg/kg in 100 μl of sterile saline solution) daily starting on the day preceding the infection and finishing a day before the desired time points (see below). The dose causes 85% inhibition of the lipopolysaccharide-induced increase in total plasma nitrite/nitrate concentration and is safe for mice [48]. Control mice received the sterile saline solution only and were infected in the same manner. At the desired time points (18 hpi for pinnae samples, 3 and 7 dpi for CNS samples), both control and AG-treated mice were anaesthetized by isoflurane and bled out. Individual pinnae or spinal cords and cerebella were extracted and separately torn apart by sharp forceps in the PBS. Schistosomula were then collected under a stereomicroscope, counted, fixed with hot water and measured (Quick Photo Micro 3.0).

Additionally, the effect of AG treatment on the nervous tissue myelination was examined 0 and 7 dpi. Spinal cords of control and AG treated mice were processed for immunohistochemistry following the aforementioned protocol. Rabbit anti-mouse MBP polyclonal antibody (1:1000, Thermo Fisher Scientific) was used and sucrose antigen retrieval was not performed for this staining.

### Peptidase activity of recombinant *T. regenti* cathepsins B after treatment by NO/ONOO^−^ donors

The lyophilized recombinant *T. regenti* cathepsin B1.1 and B2 (rTrCB1.1 and rTrCB2, respectively), produced in *Pichia pastoris* and purified as already published [36, 43], were received from H. Dvořáková and L. Mikeš (Department of Parasitology, Charles University, Prague, Czechia). After reconstitution in 0.1 M acetate buffer (pH 5.0), the concentration of the cathepsins was measured by Quant-iT Protein Assay Kit (Thermo Fisher Scientific). Their peptidase activity was then examined by hydrolysis of the synthetic fluorogenic substrate Z-Phe-Arg-AMC (Bachem, Bubendorf, Switzerland) as described before [36, 43, 44].

In particular, 0.3 µg of the cathepsin in 0.1 M acetate buffer (pH 5.0) was mixed with 100 µl of the substrate (final concentration 25 µM) to form the total reaction mix volume of 200 µl. To examine the effect of NO and ONOO^−^ on cathepsin peptidase activity, the cathepsins were preincubated with NOR-3 (10, 1 and 0.1 μM; Cayman Chemical) and SIN-1 (10, 1 and 0.1 μM; Cayman Chemical), both dissolved in dimethyl sulfoxide (DMSO), for 30 min prior to addition of the substrate. As a positive control of cathepsin peptidase activity inhibition, the samples were pre-treated by 20 µM E-64 (an irreversible inhibitor of papain-like cysteine peptidases; Sigma-Aldrich) for 30 min. Mock controls (DMSO instead of the donor) and background controls (acetate buffer instead of the enzyme) were also included.

The experiment was performed in 96-well black flat-bottom plates (Nunc, Thermo Fisher Scientific) at room temperature. The release of fluorogenic AMC from the substrate was measured by Infinite M200 fluorometer (Tecan, Männedorf, Switzerland) at 24 °C in a 30 min kinetic cycle (2-min intervals). The increase of relative fluorescence units (RFU) in time (∆RFU/min) was assessed and the activity of each sample was expressed in relation to the untreated group [44].

### *In vitro* preparation of schistosomula and treatment by NO/ONOO^−^ donors

Schistosomula were prepared by mechanical *in vitro* transformation as described before [46, 49]. Briefly, freshly collected cercariae were concentrated on ice, washed with sterile PBS and passed through the 23G-syringe needle 20 times to detach the tails. After washing, the parasites were grown in the schistosome cultivation medium (SCM; prepared according to Basch [50], using 1% (w/v) BSA instead of serum) for 18 h at 37 °C and 5% CO_2_ and proceeded for treatment by NO or ONOO^−^ donors.

Schistosomula were treated by NOR-5 (0.1 and 0.5 mM; Enzo Life Sciences, Farmingdale, USA) and SIN-1 (1.5 and 3 mM), which release NO and ONOO^−^, respectively. Thirty schistosomula per well were treated in a 24-well plate with 0.5 ml of the medium for 48 h. NOR-5-treated schistosomula were cultivated in SCM, but for those exposed to SIN-1, the medium was changed to M199 (Thermo Fisher Scientific) supplemented by 0.1% (w/v) glucose, 1% (w/v) BSA and antibiotic-antimycotic solution (100 U/ml penicillin, 0.1 mg/ml streptomycin, 0.25 μg/ml amphotericin B; Sigma-Aldrich). This was necessary to prevent unwanted production of hydrogen peroxide resulting from the interaction between HEPES (present in SCM) and SIN-1 [51]. Both NOR-5 and SIN-1 were dissolved directly in the respective medium. Untreated schistosomula were used as a negative control group.

### Assessment of schistosomula viability after treatment by NO/ONOO^−^ donors

Two approaches, methylene blue staining [52] and quantification of lactate production [53], were employed to concurrently evaluate the viability of schistosomula after NOR-5 or SIN-1 treatment.

Medium samples (100 μl) were collected and filtered through the Amicon Ultra-0.5 Centrifugal Filter Unit, 10 kDa (Merck Millipore). The concentration of lactate in the deproteinized samples (diluted 1:49 in the lactate assay buffer) was measured by fluorometric l-Lactate Assay Kit (Abcam) according to the manufacturer’s instructions. The experiment was performed in 96-well black flat-bottom plates (Thermo Fisher Scientific) and the fluorescence release was measured by Infinite M200 fluorometer (Tecan) using 535/590 nm excitation/emission filters. The concentration of lactate was calculated according to the standard curve.

Schistosomula remaining in the wells were rinsed in PBS and stained 10 min by 0.15% (w/v) methylene blue, which was added 1:1 (final concentration 0.075%). After washing in PBS, the schistosomula were examined under the stereomicroscope. Schistosomula with more than half of the body stained in blue were considered dead.

Additionally, motility of NOR-5-treated schistosomula was analyzed by the open-source ImageJ plugin wrMTrck (www.phage.dk/plugins/wrmtrck.html, [54, 55]). Specifically, the number of individual schistosomula body contractions was enumerated in 30-s movies captured by the camera (DP70, Olympus, Tokyo, Japan) attached to the inverted microscope (IX50, Olympus, Tokyo, Japan).

### Examination of NO/ONOO^−^-treated schistosomula by electron microscopy

Schistosomula dedicated to examination by scanning (SEM) or transmission (TEM) electron microscopy were rinsed 3 times for 10 min in 50 mM HEPES washing buffer (pH 7.0, osmolality 280 mOsm/kg adjusted by NaCl), fixed in 2.5% glutaraldehyde in HEPES washing buffer overnight at 4 °C and then post-fixed with 4% osmium tetroxide in HEPES washing buffer in 1:1 ratio (final concentration 2%) for 1 h at room temperature. After excessive washing 3 times for 10 min by HEPES washing buffer, the schistosomula were dehydrated in the increasing ethanol series (30, 50, 70 and 80% ethanol, each step for 10 min; 90, 96 and 3× 100% ethanol, each step for 5 min). Dehydration was terminated by 100% acetone 3 times for 5 min.

In the end, schistosomula for SEM were dried by critical point dryer (CPD 030, Bal-Tec, Pfäffikon, Switzerland), coated with gold using a sputter coater (SCD 050, Bal-Tec, Pfäffikon, Switzerland) and observed by SEM JEOL 6380 LV. Schistosomula for TEM were embedded into Spurr epoxy resin (Sigma-Aldrich) through increasing acetone:Spurr solution series (3:1 for 2 h, 1:1 for 4 h, 1:3 overnight, pure Spurr for 12 h) and after final centrifugation in Beem capsules, the samples were polymerized in fresh Spurr for 48 h in 60 °C. Afterwards, thin sections (60–70 nm) were prepared onto standard copper electron microscopy grids and observed by TEM (JEOL 1011, JEOL, Tokyo, Japan).

### Statistical analyses

Mann-Whitney test was used to evaluate differences between two groups, while one-way analysis of variance (ANOVA) followed by Šidák’s or Dunnett’s test was applied for comparison of more than two groups. Brown-Forsythe ANOVA and Dunnett’s T3 test were employed for comparison of groups with unequal standard deviations and Kruskal-Wallis test was applied if data were not distributed normally. To examine the effects of both time and treatment, two-way ANOVA followed by Šidák’s test was used. Categorical data were assessed by Fisher’s exact test. The analyses were performed in GraphPad Prism (version 8.3) and *P*-values < 0.05 were considered significant. Whiskers in box-and-whisker plots indicate minimum/maximum values.

## Results

### *Trichobilharzia regenti* triggered NO production in the skin and 3-NT formation in the spinal cord

**Fig. 1:**
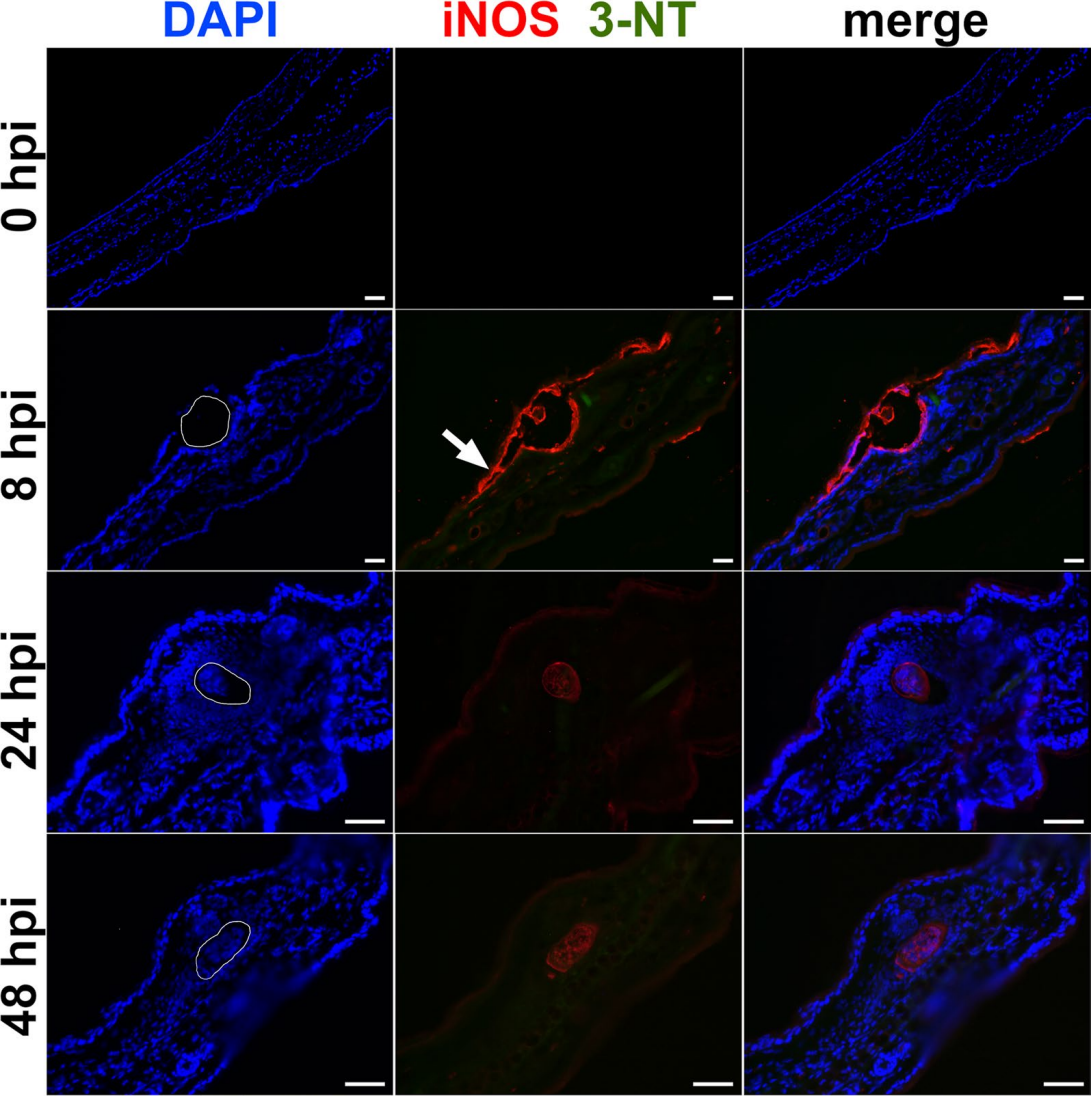
Immunolocalization of inducible nitric oxide synthase (iNOS) a 3-nitrotyrosine (3-NT) in pinnae of mice infected with *Trichobilharzia regenti*. The iNOS signal (red) surrounded the penetrating schistosomula 8 h post-infection (hpi) and spread also in the adjacent epidermal area (white arrow). Leukocytes infiltrated the lesion 24 hpi, but neither iNOS nor 3-NT signals were detected at this or later (48 hpi) time points. The space occupied by schistosomula is marked with white line in DAPI images. Three mice per time point and at least two schistosomula-positive slides per mouse were analyzed. The faint red signal observed 24 and 48 hpi represents a non-specific binding of the rabbit polyclonal antibody to the schistosomula and was observed also in control samples incubated with negative rabbit serum (not shown). *Scale-bars*: 50 μm

We first assessed the presence of iNOS and 3-NT in the skin and the spinal cord which are affected by *T. regenti* migration. The parasite successfully entered the skin and schistosomula were detected in the upper layers of epidermis 8 hpi. A strong iNOS signal was observed in the epidermal cells around 80% of schistosomula and also in the adjacent skin superficial area (Fig. 1). Later, schistosomula moved to the dermis and were surrounded by massive clusters of infiltrating leukocytes 24 and 48 hpi, but no iNOS signal was detected (Fig. 1). Considering 3-NT, no signal was noticed in the skin at any of the examined time points.

**Fig. 2:**
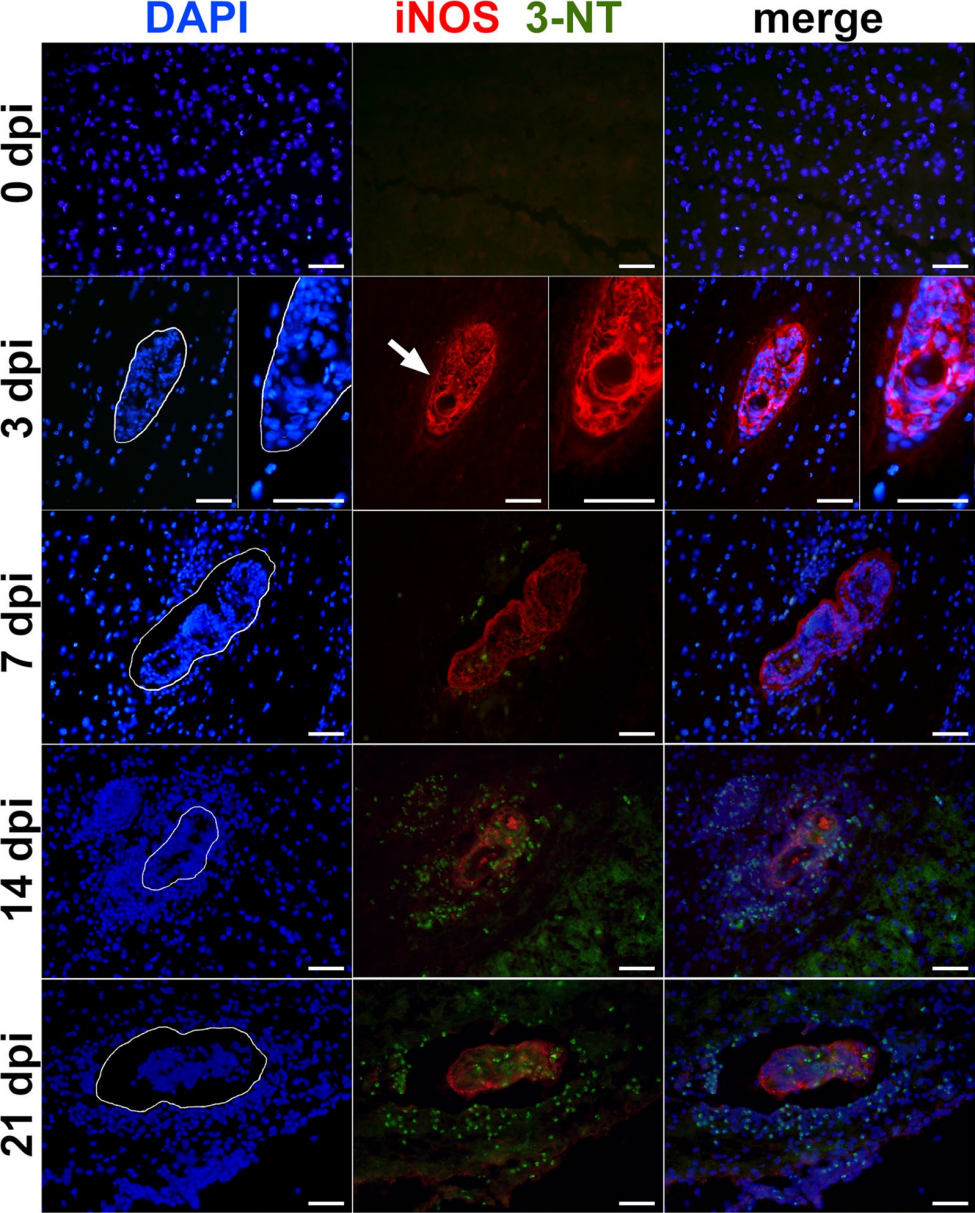
Immunolocalization of inducible nitric oxide synthase (iNOS) a 3-nitrotyrosine (3-NT) in the spinal cord of mice infected with *Trichobilharzia regenti*. The iNOS signal (red, pointed by white arrow) surrounded schistosomula 3 days post-infection (dpi). The signal of 3-NT (green) started to appear 7 dpi and was more frequent 14 and 21 dpi. It was localized within the cells surrounding the schistosomula as well within the schistosomula internal tissues. The space occupied by schistosomula is marked with white line in DAPI images. Three mice per time point and at least two schistosomula-positive slides per mouse were analyzed. Similarly to Fig. 1, the non-specific binding of the rabbit polyclonal anti-iNOS antibody to the schistosomula was observed. *Scale-bars*: 50 μm

In the spinal cord, the iNOS signal was localized in the vicinity of 60% of schistosomula 3 dpi. However, it was slightly weaker than in the skin and it vanished before 7 dpi (Fig. 2). On the contrary, the 3-NT signal started to appear in the nervous tissue close to schistosomula 7 dpi and was most prominent in 90% of the samples from 14 and 21 dpi. It exhibited (peri) nuclear distribution within the cells surrounding the schistosomula (Fig. 2). Furthermore, a rising proportion of 3-NT-positive schistosomula was observed as the infection proceeded. Specifically, 14% and 44% of schistosomula contained 3-NT signal 7 and 21 dpi, respectively. A summary and statistical evaluation of iNOS and 3-NT signal frequencies at different time points is provided in Additional file 1: Table S1.

The concentration of serum nitrites/nitrates did not change significantly throughout the infection (Additional file 2: Figure S1).

### iNOS inhibition moderately increased *T. regenti* burden in the spinal cord 7 dpi and ambiguously affected parasite growth

**Fig. 3:**
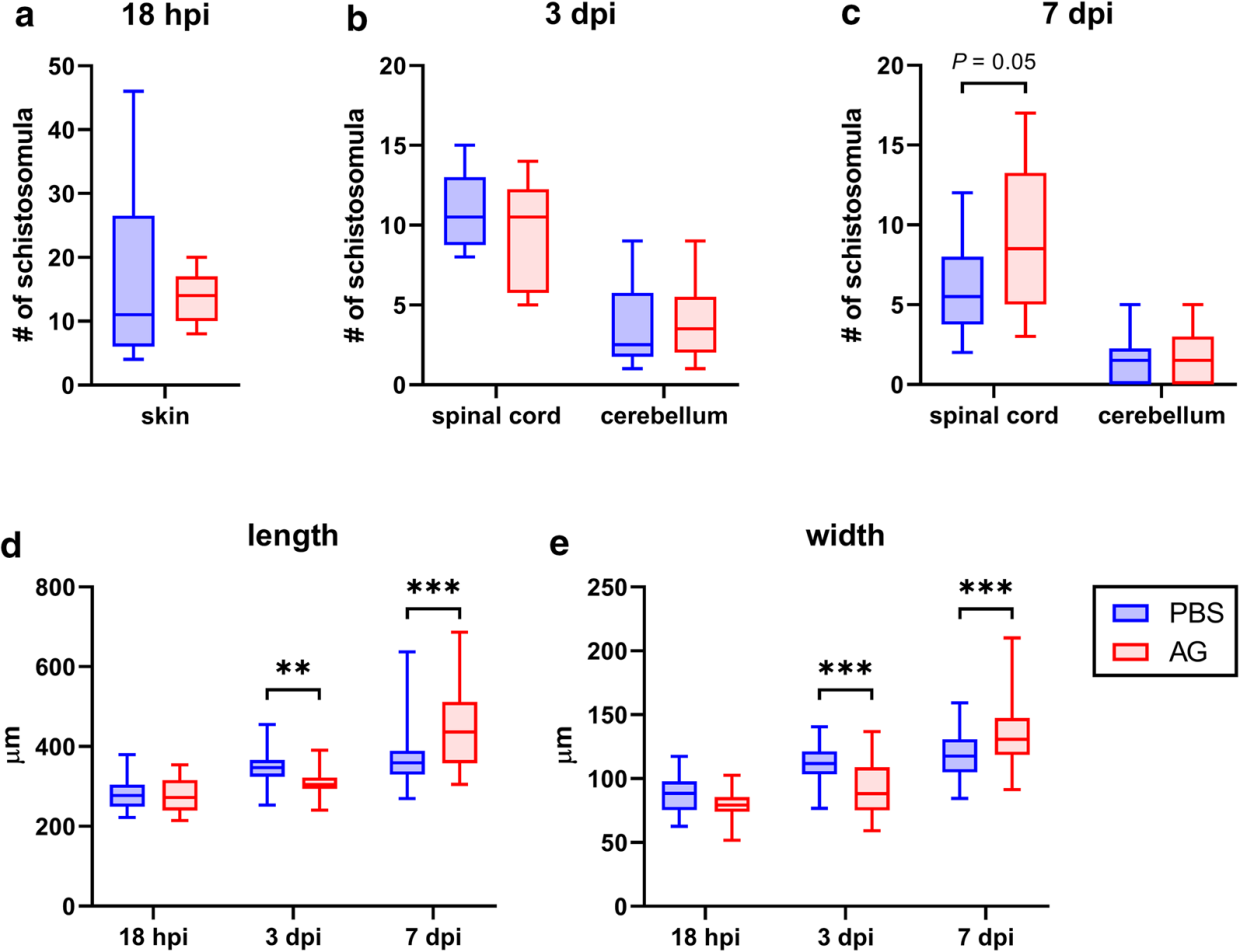
Effects of inducible nitric oxide synthase (iNOS) inhibition by aminoguanidine (AG) on *Trichobilharzia regenti* infection in mice. No effect on parasite burden was observed 18 h post-infection (hpi) in the skin (**a**) and 3 days post-infection (dpi) in the spinal cord (**b**). However, moderately increased parasite burden was noticed in the spinal cord, but not cerebellum of AG-treated mice (red boxplots) 7 dpi in comparison with the control group treated by phosphate-buffered saline (PBS; blue boxplots) (**c**). The graphs **a-c** show pooled data from two experiments, each using five mice per group. Schistosomula isolated from AG-treated mice were shorter (**d**) and thinner (**e**) than those from control mice 3 dpi, but the opposite was observed 7 dpi. For each time point, 36–56 schistosomula fixed in hot water were measured. Data were evaluated by Mann-Whitney test (**a**) or two-way ANOVA followed by Šidák’s test (**b**–**e**); **P < 0.01, ***P < 0.001

To evaluate the role of NO in control of *T. regenti* infection, we inhibited iNOS by AG and examined the mice 18 hpi, 3 and 7 dpi. Early after the infection, control and AG-treated mice had similar parasite burden (i.e. number of schistosomula) in the pinnae 18 hpi (Fig. 3a) and spinal cords or cerebella 3 dpi (Fig. 3b). Later, 7 dpi, most of the schistosomula were localized in the spinal cord in control mice and only a few of them reached the cerebellum (Fig. 3c, blue boxplots). However, moderately increased parasite burden was observed in the spinal cord of AG-treated mice (Šidák’s multiple comparisons test: *P* = 0.0521) but the effect was absent in the cerebellum (Fig. 3c, red boxplots).

Considering the size, schistosomula from AG-treated mice were comparable to those from control mice 18 hpi, but were surpassed by the latter 3 dpi (Šidák’s multiple comparisons test, length: *P* = 0.0046, width: *P* < 0.0001) (Fig. 3d, e). On the contrary, significantly larger (Šidák’s multiple comparisons test, length: *P* < 0.0001, width: *P* = 0.0003) schistosomula were found in AG-treated mice 7 dpi (Fig. 3d, e).

AG treatment did not alter schistosomula localization within the spinal cord tissue, their distribution in spinal cord segments or demyelination of adjacent nervous tissue (Additional file 3: Figure S2).

### NO decreased activity of *T. regenti* essential peptidases *in vitro*

As the previous results indicated different parasite burden and schistosomula size in AG-treated mice, we tested the effect of NO and ONOO^−^ on the activity of rTrCB1.1 and rTrCB2, essential histolytic and digestive peptidases of *T. regenti*.

**Fig. 4:**
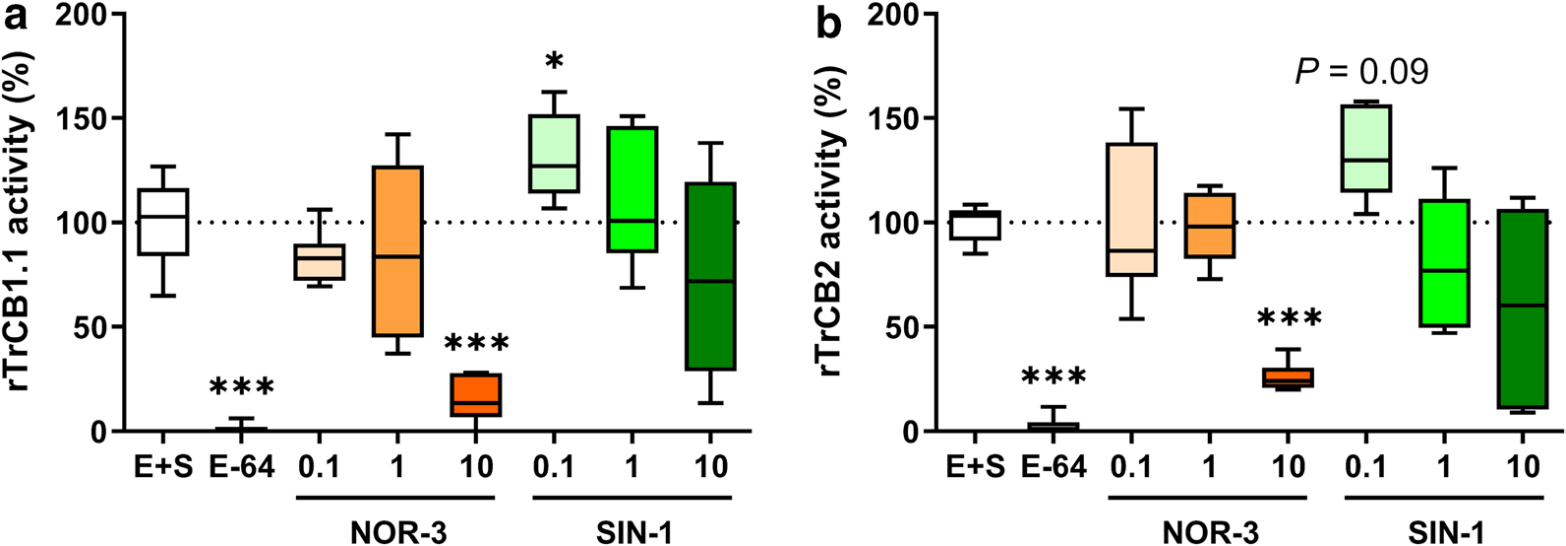
Activity of *Trichobilharzia regenti* cysteine peptidases after the treatment by nitric oxide and peroxynitrite. *T. regenti* cathepsins B1.1 (**a**) and B2 (**b**) were *in vitro* incubated with a fluorogenic substrate (E+S, the reference sample), 20 µM E-64 (the inhibitor of cysteine peptidases) and 0.1–10 μM NOR-3 (the donor of nitric oxide) or SIN-1 (the donor of peroxynitrite). The activity of both cathepsins was inhibited by treatment with 10 μM NOR-3 but enhanced after treatment with 0.1 μM SIN-1. The graphs show pooled data from three experiments, each performed in triplicates. Data were evaluated by Brown-Forsythe ANOVA and Dunnett’s T3 test; *P < 0.05, ***P < 0.001

Incubation with 10 μM NOR-3, the NO donor, significantly decreased the peptidase activity of rTrCB1.1 and rTrCB2 to 15.8 ± 10.5% and 26.1 ± 6.6% (both Dunnett’s T3 multiple comparisons tests: *P* < 0.0001), respectively (Fig. 4, orange boxplots). In both cases, the peptidase activity negatively associated with the actual concentration of nitrites, degradation products of NO released from NOR-3 (Additional file 4: Figure S3).

As for SIN-1, the ONOO^−^ donor, an increase of rTrCB1.1 peptidase activity to 130.9 ± 19.9% (Dunnett’s T3 multiple comparisons tests: *P* = 0.0204) was noticed after incubation with 0.1 μM SIN-1; the similar trend was also observed in the case of rTrCB2 (Dunnett’s T3 multiple comparisons tests: *P* = 0.0898) (Fig. 4, orange boxplots). Incubation of both cathepsins with higher concentrations of SIN-1 suggested declining of the peptidase activity but the results were rather variable and non-conclusive.

### ONOO^−^, but not NO, reduced viability and damaged ultrastructure of *T. regenti* schistosomula *in vitro*

To examine the direct effects of RNS on schistosomula, they were cultivated with NOR-5 and SIN-1, donors of NO and ONOO^−^, respectively. After 48 h, their viability was evaluated as well as changes in topography and ultrastructure.

**Fig. 5:**
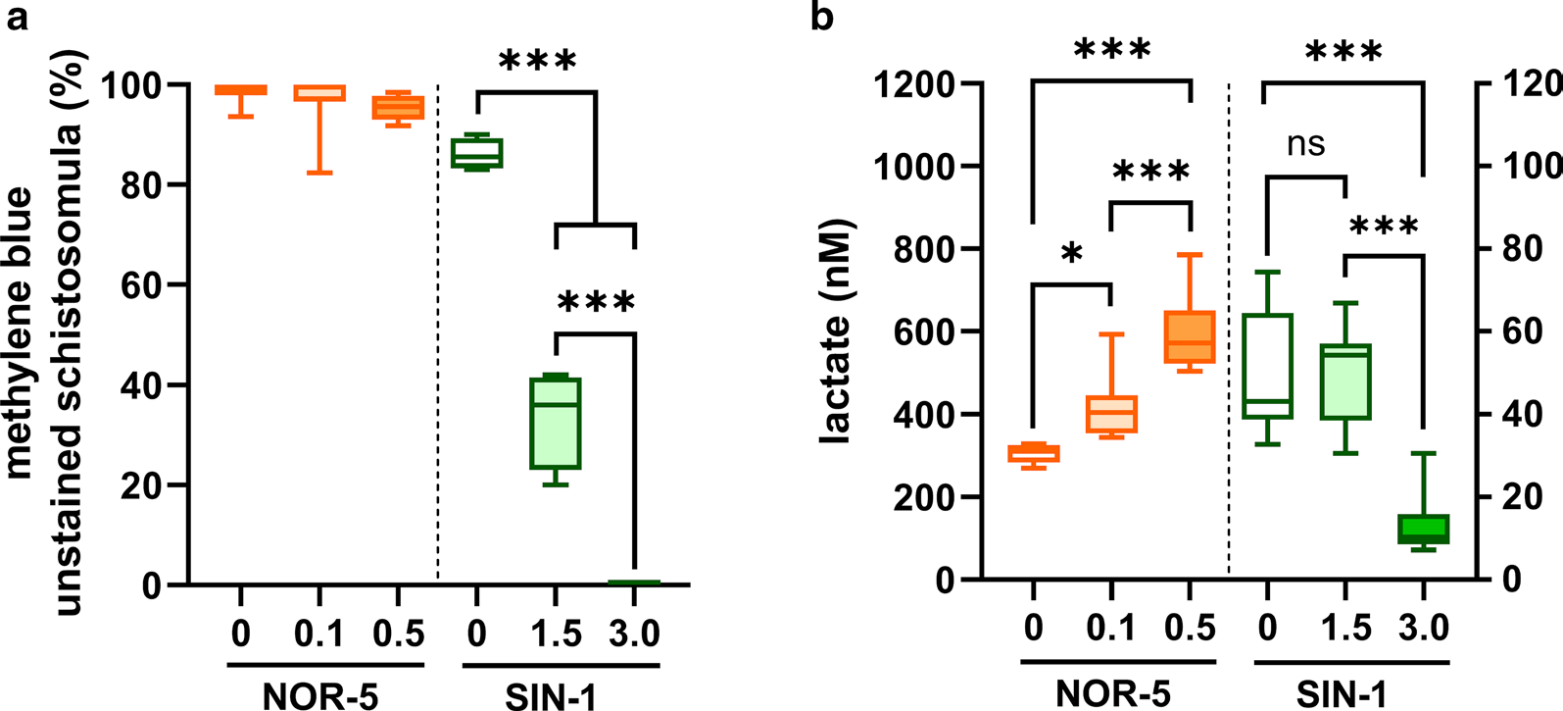
Viability of *Trichobilharzia regenti* schistosomula after 48-h treatment by donors of nitric oxide or peroxynitrite. Nitric oxide was released from NOR-5 (0.1 and 0.5 mM) and peroxynitrite from SIN-1 (1.5 and 3 mM). The viability was assessed by methylene blue staining (**a**) and analysis of lactate production (**b**). NOR-5 treated schistosomula (orange boxplots) remained mostly unstained, i.e. viable, but they increased production of lactate in a dose-dependent manner. However, schistosomula treated by SIN-1 (green boxplots) evinced decreased viability as well as reduced production of lactate. The graphs show pooled data from three experiments, each performed in quadruplicates. Data were evaluated by one-way ANOVA followed by Šidák’s test; *P < 0.05, ***P < 0.001

Treatment of schistosomula by NOR-5 did not alter their viability (Fig 5a, orange boxplots), but it increased their production of lactate in a dose-dependent manner (Fig. 5b, orange boxplots; ANOVA: *F*_(2, 21)_ = 32.28, *P* < 0.0001). The actual concentration of nitrites, degradation products of NO released from NOR-5, present in the medium are provided in Additional file 5: Figure S4. No changes in schistosomula motility were noticed (Additional file 6: Figure S5). On the contrary, treatment by 1.5 mM SIN-1 reduced schistosomula viability to 35.5 ± 9.9% and 3 mM SIN-1 always killed all of them (Fig. 5a, green boxplots; ANOVA: *F*_(2, 9)_ = 205.6, *P* < 0.0001). No changes in lactate production were noticed in 1.5 mM-treated schistosomula, but it was significantly diminished after treatment by 3 mM SIN-1 (Fig. 5b, green boxplots; ANOVA: *F*_(2, 32)_ = 38.86, *P* < 0.0001).

**Fig. 6:**
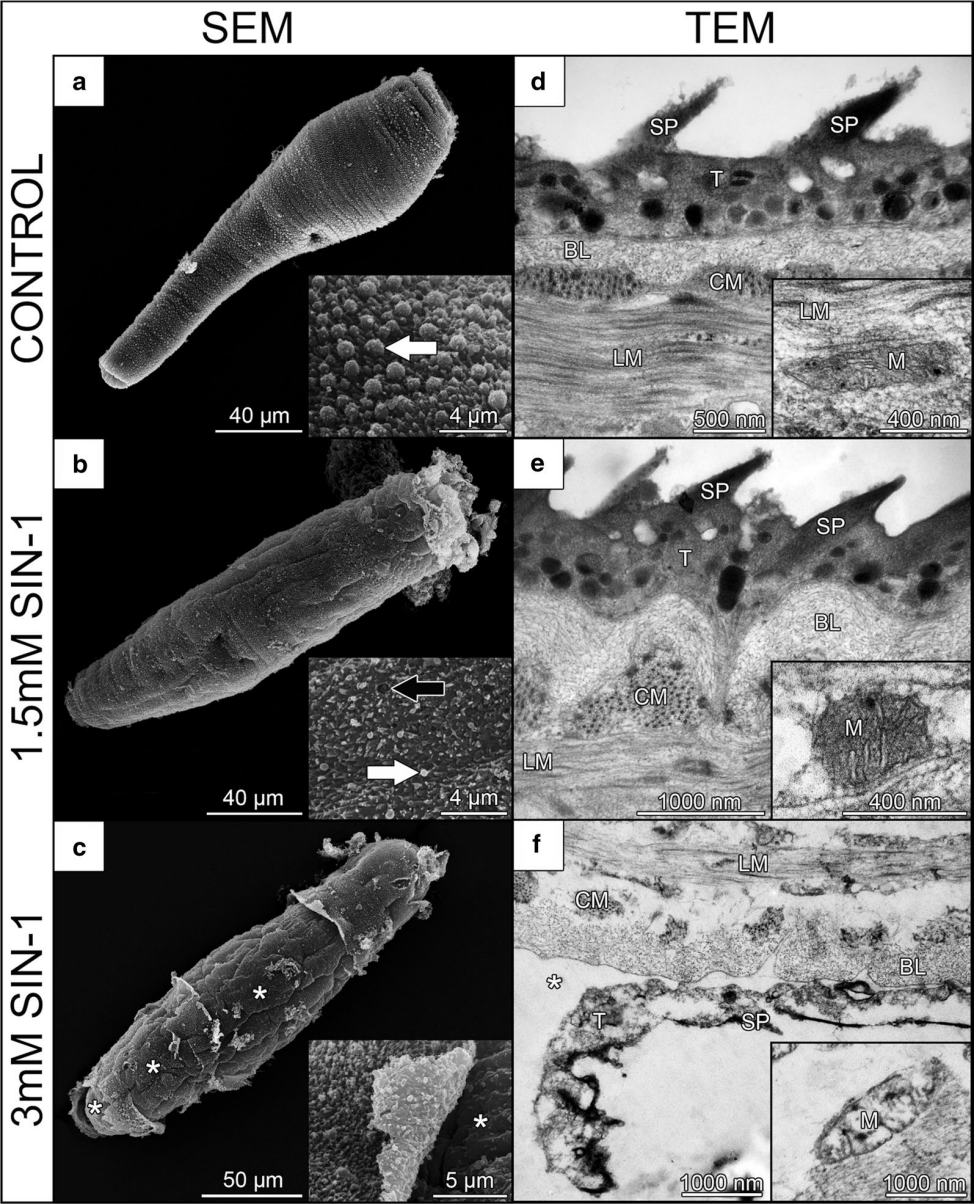
Ultrastructural changes of *Trichobilharzia regenti* schistosomula treated with SIN-1, the donor of peroxynitrite. The schistosomula were treated for 48 h and then examined under scanning (SEM; **a-c**) and transmission (TEM; **d**–**f**) electron microscopy. As revealed by SEM, the tegument of 1.5 mM SIN-1-treated schistosomula had fewer blebs (white arrows) covering the layer of spines than the tegument of the control group. Also, the topography of tegumental spines was changed after 1.5 mM SIN-1 treatment and small holes (black arrow) appeared in the surface (**b**). No differences were observed between these groups in TEM (**d**, **e**) including ultrastructure of mitochondria (insets in **d**, **e**). Schistosomula treated with 3 mM SIN-1 had severely destructed tegument partially revealing the basal lamina (white asterisks) (**c**, **f**). Subtegumental mitochondria had disrupted inner membranes and cristae (inset in **f**). *Abbreviations*: T, tegument; SP, spines; BL, basal lamina; CM, circular muscles; LM, longitudinal muscles; M, mitochondria

The effect of NO and ONOO^−^ on schistosomula was also examined by SEM and TEM. While NOR-5 treatment did not cause any ultrastructural or surface changes (Additional file 7: Figure S6), remarkable alterations were observed after cultivation of schistosomula with SIN-1 (Fig. 6). Small holes appeared in the surface tegument and the tegumental spines were disordered and more submerged in schistosomula treated by 1.5 mM SIN-1 than in control ones. Furthermore, blebs usually found on the tips of the spines were less numerous in this group (Fig. 6a, b). Surprisingly, these defects were not noticed in TEM examination (Fig. 6d, e).

Apart from the minor surface alterations seen in 1.5 mM SIN-1-treated group, severe tegumental disruption was observed in schistosomula treated with 3 mM SIN-1. In particular, the tegument was locally cracked and partially or fully peeled off uncovering the underlying basal lamina (Fig. 6c). Sometimes even the structure of circular muscle fibres, localized beneath the basal lamina, emerged in distinct stripes. TEM observation revealed vacuolization, disintegration of the tegument and its separation from the basal membrane (Fig. 6f). Furthermore, loss of integrity and electron density was evident in internal tissues. Also, subtegumental mitochondria were damaged exhibiting vacuolization, disruption of inner membranes and distortion of cristae (Fig. 6f, inset).

## Discussion

Avian schistosomes cannot survive in mammals [56–58], but the mechanisms involved in infection control are unknown. Here we examined the role of NO in the immune response of mice experimentally infected with *T. regenti*. It is the prevailing model species of avian schistosomes and the causative agent of human cercarial dermatitis [34] distinguished also for its neuropathogenicity [37, 41].

Skin is the first barrier that *T. regenti* must breach. Neutrophils are recruited into the dermis 4–6 hpi, eosinophils and macrophages appear 18–20 h later [37]. Corroborating with these findings, we observed massive infiltration of leukocytes enclosing the schistosomula in pinnae 24 and 48 hpi. A similar reaction, accompanied by increased expression of iNOS, was also noticed in the skin of *S. mansoni*-infected mice immunized by attenuated cercariae [59]. Although the recruited leukocytes are commonly capable of NO production, Ramaswamy et al. [59] assumed that rather the skin resident cells produced NO. This assumption is consistent with our direct observation of iNOS exclusively in the upper layers of the epidermis 8 hpi, suggesting that keratinocytes produced NO early after the infection. It is likely a non-specific reaction to the parasite-driven skin injury, which might improve the healing process [60, 61]. However, a specific action of parasite excretory-secretory products (ESP) released during penetration cannot be entirely excluded since *S. mansoni* cercarial ESP did trigger pro-inflammatory cytokines in murine keratinocytes [62].

NO produced in the epidermis early after penetration could hinder further parasite migration by affecting its energy metabolism. Although detrimental effects of NO on aerobic respiration were reported for human schistosomes [11, 14], we did not observe decreased viability or mitochondrial damage, a usual ultrastructural footprint of NO-related disruption of aerobic respiration, in *T. regenti* schistosomula treated by NO *in vitro*. This led us to assume that NO has no detrimental effects on *T. regenti* skin schistosomula aerobic metabolism. Indeed, it agrees with previous studies showing that schistosomula shift from fully aerobic to mostly anaerobic energy metabolism soon after the penetration and secrete lactate as the major metabolic end-product [16, 63–65]. This transition is triggered and largely dependent on the sudden availability of glucose in the host tissues [66]. However, we observed an increased, dose-dependent production of lactate by schistosomula cultivated in the glucose-rich medium after they were treated by NO donors. Similarly, the bacterium *Staphylococcus aureus* was shown to switch to massive lactate production after NO treatment to maintain redox homeostasis during nitrosative stress [67]. Thus, we hypothesize that NO might contribute to the schistosomula metabolic transition by inhibiting respiration [11, 14, 68, 69] and promoting anaerobic metabolism associated with increased production of lactate [70].

NO has also emerged as an important factor driving the development and neuronal functions of invertebrates, including platyhelminths [71, 72]. Such a need for “NO developmental trigger” could explain our unexpected observations of retarded schistosomula growth in the early phase of the infection in AG-treated mice. However, further research is needed to identify the source of NO responsible for this phenomenon. It could be the host iNOS expressed by keratinocytes but also NO produced by the parasite itself. Indeed, NO synthase activity was demonstrated in human schistosomes and its role in developmental processes was suggested [73–75], but there has been no further progress in this field. Similarly, relevant data are currently not available for *T. regenti*, as well as long-term *in vitro* cultivation techniques, which would facilitate validation of these hypotheses.

Apart from energy metabolism and development, NO can also target cysteine peptidases, vital enzymes contributing to parasite pathogenicity and virulence [76, 77]. For example, NO inhibited cysteine peptidases of the protozoan parasites *Plasmodium falciparum*, *Leishmania infantum* or *Trypanosoma cruzi in vitro* [78]. However, the relevance of this effect was not evaluated *in vivo*. Here we first demonstrated the inhibitory effect of NO on rTrCB1.1 and rTrCB2 peptidase activity *in vitro*. Cathepsins B are essential peptidases of *T. regenti* playing crucial roles in the invasion of host tissues (such as TrCB2 degrading collagen, elastin, keratin and MBP; [36]) and also digestion (such as TrCB1.1 degrading collagen and MBP; [43, 44]). Consequently, their impairment by NO can impact on the course of *T. regenti* infection.

Supporting this assumption, we observed a moderately increased parasite burden in the spinal cord of mice with inhibited iNOS 7 dpi. It could be explained by higher (NO-unaffected) activity of TrCB2 which helped schistosomula to cleave host proteins more effectively, hence enabling faster migration within the CNS. Furthermore, the schistosomula from these mice were significantly larger, suggesting that inhibition of iNOS might have promoted parasite growth in this period. It could be associated with improved (NO-unaffected) function of TrCB1.1 which enables schistosomula to feed on myelin [41, 45], i.e. to better digest MBP and gain more nutrients.

Our observations of NO-mediated disruption of proteolytic machinery in *T. regenti* schistosomula raise the question whether it can happen also in human schistosomes. As for them, the major attention has been paid especially to the NO-related inhibition of schistosomula aerobic metabolism [11, 13, 15, 16]. Other mechanisms, including the effect of NO on cysteine peptidases, have not been evaluated yet even though these enzymes play a crucial role in schistosome biology [77, 79]. This might be due to the generally accepted view that the host immunity against schistosomes shifts from initial Th1 (targeting schistosomula aerobic metabolism by NO) to Th2/Treg which are not associated with NO production harmful to adults [80, 81]. However, their vital enzymes prone to NO-mediated inactivation might be suitable drug targets. This is the case of hybrid therapeutic compounds consisting of praziquantel and NO-donors furoxans which significantly inhibited *S. mansoni* thioredoxin glutathione reductase *via* (seleno) cysteine nitrosylation. This enzyme is required for maintaining the redox homeostasis and its inhibition led to impaired motility or even death of *S. mansoni* adults *in vitro* [20, 82].

ONOO^−^ is more reactive than NO and severely harms helminths *in vitro* [83, 84]. Accordingly, treatment of *T. regenti* schistosomula by 3 mM SIN-1, the donor of ONOO^−^, significantly decreased their viability and devastated the tegument and mitochondria. However, the effects of 1.5 mM SIN-1 were milder and caused only a minor alteration of the tegument, specifically, partial loss of the blebs on the tips of the tegumental spines. Under physiological conditions, such blebs are regularly distributed on the surface of early schistosomula. This is a manifestation of the ongoing cercaria-schistosomulum transformation during which the old membrane is discarded in the form of small microvilli [85, 86]. Hence, the loss of these blebs after treatment by 1.5 mM SIN-1 might be a sign of inhibited surface transformation *in vitro*.

Although ONOO^−^ strongly impaired *T. regenti* schistosomula *in vitro*, the relevance of these effects *in vivo* must be interpreted with caution. Actually, our observations rather do not support the view that ONOO^−^ participates in *T. regenti* elimination in mice at the examined time points. First, we did not detect any 3-NT concurrently with iNOS which would point to the formation of ONOO^−^ possibly acting against the schistosomula [4]. Secondly, no ultrastructural changes, resembling those observed by us after *in vitro* treatment by SIN-1, were noticed in schistosomula directly in the infected spinal cord [87]. However, the actions of ONOO^−^ beyond the examined time points/period cannot be fully excluded. Anyway, other mechanisms causing nitrosative stress must have been engaged in the spinal cord since 3-NT was markedly present in the spinal cord 14 and 21 dpi when no iNOS was detected. At these time points, the schistosomula are enclosed by massive clusters of microglia and macrophages [41] and granulocyte infiltration peaks in the spinal cord [88]. Thus, we hypothesize that protein tyrosine nitration was mediated by a myeloperoxidase-dependent pathway which converts extracellular nitrites to nitrating species and is present in the aforementioned cell types [89–91].

## Conclusions

Overall, this study shows the role of NO in the immune response of mice infected with the neuropathogenic schistosome *Trichobilharzia regenti*, a causative agent of human cercarial dermatitis. It seems that NO has ambiguous effects: it promotes the parasite growth in the early phase of the infection but prevents it later, which is also accompanied by suspended migration in the CNS. These effects likely stem from continuous and chronic debilitation of the parasite, partly related to NO inactivation of its vital peptidases, rather than from acute NO cytotoxicity.

## Supplementary information

**Additional file 1: Table S1.** Summary of iNOS and 3-NT signal occurrence around *Trichobilharzia regenti* schistosomula in the skin and the spinal cord.

**Additional file 2: Figure S1.** Concentrations of serum nitrite/nitrates during infection of mice with *Trichobilharzia regenti*.

**Additional file 3: Figure S2.** Effects of inducible nitric oxide synthase (iNOS) inhibition by aminoguanidine (60 mg/kg i.p.) on *Trichobilharzia regenti* infection in mice 7 days post-infection.

**Additional file 4: Figure S3.** A negative association between the activity of *Trichobilharzia regenti* cathepsin B1 or B2 and amount of nitric oxide released from NOR-3.

**Additional file 5: Figure S4.** Amount of nitric oxide released from NOR-5 shown as the concentration of nitrites in the medium.

**Additional file 6: Figure S5.** Number of body contractions performed by *T. regenti* schistosomula treated *in vitro* by NOR-5, the donor of NO.

**Additional file 7: Figure S6.** Ultrastructural changes of *Trichobilharzia regenti* schistosomula treated by 0.5mM NOR-5 visualised by scanning and transmission electron microscopy.

## Data Availability

All data supporting the conclusions of this article are included within the article and its additional files.

## Abbreviations

(r)TrCB1.1: (recombinant) *Trichobilharzia regenti* cathepsin B1.1
(r)TrCB2: (recombinant) *Trichobilharzia regenti* cathepsin B2
3-NT: 3-nitrotyrosine
AG: aminoguanidine hydrochloride
ANOVA: analysis of variance
BSA: bovine serum albumin
DMSO: dimethyl sulfoxide
dpi: day(s) post-infection
ESP: excretory/secretory products
hpi: hour(s) post-infection
iNOS: inducible nitric oxide synthase
NO: nitric oxide
ONOO^−^: peroxynitrite
PBS: phosphate buffered saline
RFU: relative fluorescence unit
RNS: reactive nitrogen species
SCM: schistosome cultivation medium
SEM: scanning electron microscopy
TEM: transmission electron microscopy
