# Supplementary material for: Nitric oxide debilitates the neuropathogenic schistosome Trichobilharzia regenti in mice, partly by inhibiting its vital peptidases
Source: Parasit Vectors. 2020 Aug 20;13:426. doi: 10.1186/s13071-020-04279-9 (PMC7439556; doi:10.1186/s13071-020-04279-9)
Supplement: Supplementary file 1 — Additional file 1: Table S1. Summary of iNOS and 3-NT signal occurrence around Trichobilharzia regenti schistosomula in the skin and the spinal cord. [file 13071_2020_4279_MOESM1_ESM.docx]

**Additional file 1: Table S1.** A summary of iNOS and 3-NT signal occurrence around *Trichobilharzia regenti* schistosomula in the skin and the spinal cord. The values stand for the number of positive/examined slides. Three mice were infected for each timepoint and at least two schistosomula-positive slides per mouse were examined. Data were evaluated by Fisher's exact test*. Abbreviations*: 3-NT, 3-nitrotyrosine; CI, 95% confidence interval; dpi, days post infection; hpi, hours post infection; iNOS, inducible nitric oxide synthase; OR, odds ratio

| **Tissue** | **Timepoint** | **iNOS** | **3-NT** | **3-NT** |
| --- | --- | --- | --- | --- |
|  |  |  | **(host tissue)** | **(parasite tissue)** |
| **Skin** | 0 hpi | 0/6 | 0/6 | 0/6 |
|  | 8 hpi | 10/11  (P = 0.0006,  OR: 0.0109,  CI: 0.0003-0.3125) | 0/6 | 0/6 |
|  | 24 hpi | 0/6 | 0/6 | 0/6 |
|  | 48 hpi | 0/7 | 0/7 | 0/7 |
| **Spinal cord** | 0 dpi | 0/8 | 0/8 | 0/8 |
|  | 3 dpi | 13/21  (P = 0.0033,  OR: 0.0370,  CI: 0.0018-0.7286) | 0/21 | 0/21 |
|  | 7 dpi | 1/14  (P = 1,  OR: 0.5294,  CI: 0.0192-14.5600) | 11/14  (P = 0.0010,  OR: 0.0179,  CI: 0.0008-0.3949) | 2/14  (P = 0.5152,  OR: 0.2941,  CI: 0.0124-6.9300) |
|  | 14 dpi | 0/16 | 13/16  (P = 0.0002,  OR: 0.0153,  CI: 0.0007-0.3337) | 5/16  (P = 0.1304,  OR: 0.123,  CI: 0.0059-2.5420) |
|  | 21 dpi | 0/16 | 7/9  (P = 0.0023,  OR: 0.0196,  CI: 0.0009-0.4772) | 4/9  (P = 0.0824,  OR: 0.0719,  CI: 0.0032-1.6170) |
