# Supplementary material for: Nitric oxide debilitates the neuropathogenic schistosome Trichobilharzia regenti in mice, partly by inhibiting its vital peptidases
Source: Parasit Vectors. 2020 Aug 20;13:426. doi: 10.1186/s13071-020-04279-9 (PMC7439556; doi:10.1186/s13071-020-04279-9)

**Additional file 2: Figure S1.** Concentrations of serum nitrite/nitrates during infection of mice with *Trichobilharzia regenti*. No significant changes were observed. Data were evaluated by one-way ANOVA (F(4, 29) = 1.976, P = 0.1247) followed by Dunnett’s test, n=7 for each timepoint


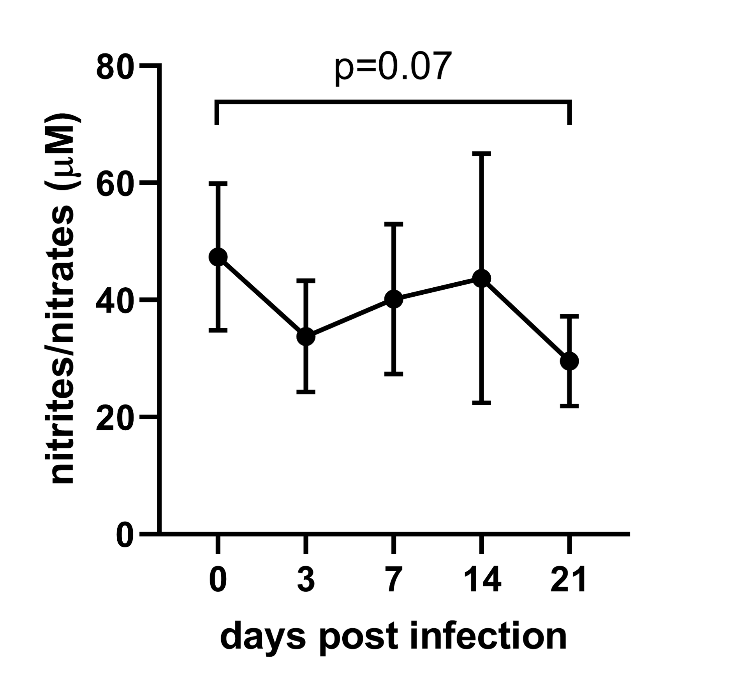

Supplement: Supplementary file 2 — Additional file 2: Figure S1. Concentrations of serum nitrite/nitrates during infection of mice with Trichobilharzia regenti. [file 13071_2020_4279_MOESM2_ESM.docx]
