# Supplementary material for: Nitric oxide debilitates the neuropathogenic schistosome Trichobilharzia regenti in mice, partly by inhibiting its vital peptidases
Source: Parasit Vectors. 2020 Aug 20;13:426. doi: 10.1186/s13071-020-04279-9 (PMC7439556; doi:10.1186/s13071-020-04279-9)

**Additional file 3: Figure S2.** Effects of inducible nitric oxide synthase (iNOS) inhibition by aminoguanidine (AG; 60 mg/kg i.p.) on *Trichobilharzia regenti* infection in mice 7 days post infection (dpi). (a) The localization of schistosomula within the spinal cord tissue where no significant differences were noticed (Fisher's exact test, P = 0.1418). Data were pooled from three mice per group. (b) The distribution of schistosomula within the spinal cord segments where no significant differences were noticed (Fisher's exact test, P = 0.3346). Data were pooled from three mice per group. (c) Mean fluorescence intensity (MFI) of myelin basic protein (MBP) signal in sections of spinal cords of control (blue bars) or AG-treated (red bars) mice 0 and 7 dpi. Two mice were used for “0 dpi” group while three mice per timepoint were analyzed for 7 dpi using at least three schistosomula-positive slides per mouse. Data are presented as mean + standard deviation; individual values for all examined slides are shown as well. Data were evaluated by two-way ANOVA followed by Šidák’s test, no significant differences were noticed (time: F(1, 50) = 0.4432, P = 0.5086; treatment: F(1, 50) = 2.624, P=0.1115). (d) Representative images of MBP staining in the spinal cord of control or AG-treated mice 7 dpi. *Scale bars* = 50 μm


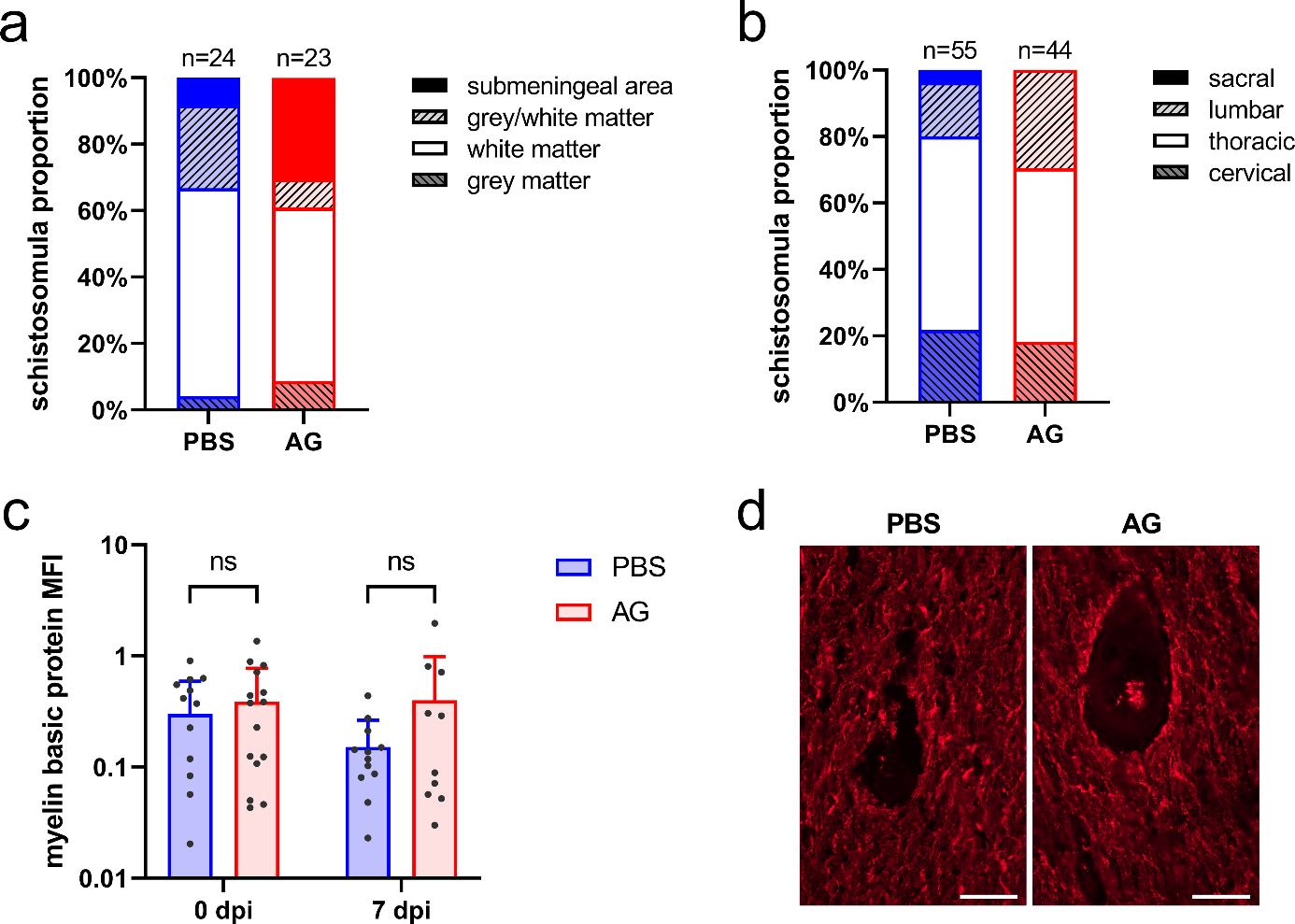

Supplement: Supplementary file 3 — Additional file 3: Figure S2. Effects of inducible nitric oxide synthase (iNOS) inhibition by aminoguanidine (60 mg/kg i.p.) on Trichobilharzia regenti infection in mice 7 days post-infection. [file 13071_2020_4279_MOESM3_ESM.docx]
