# Supplementary material for: Nitric oxide debilitates the neuropathogenic schistosome Trichobilharzia regenti in mice, partly by inhibiting its vital peptidases
Source: Parasit Vectors. 2020 Aug 20;13:426. doi: 10.1186/s13071-020-04279-9 (PMC7439556; doi:10.1186/s13071-020-04279-9)

**Additional file 4: Figure S3.** A negative association between the activity of *Trichobilharzia regenti* cathepsin B1 (rTrCB1.1, blue) or rTrCB2 (rTrCB2, red) (data from Figure 4) and amount of nitric oxide released from NOR-3. The latter is represented by the actual concentration of nitrites measured in the samples treated by 0.1, 1 and 10 μM NOR-3. The dose response model ([inhibitor] versus normalized response) was applied


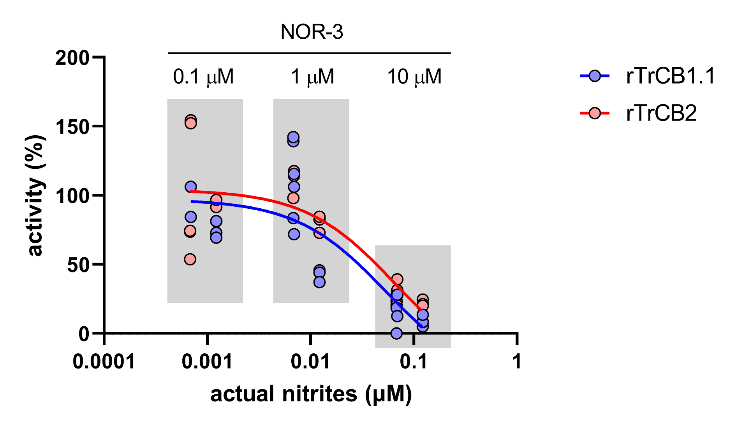

Supplement: Supplementary file 4 — Additional file 4: Figure S3. A negative association between the activity of Trichobilharzia regenti cathepsin B1 or B2 and amount of nitric oxide released from NOR-3. [file 13071_2020_4279_MOESM4_ESM.docx]
