# Supplementary material for: Nitric oxide debilitates the neuropathogenic schistosome Trichobilharzia regenti in mice, partly by inhibiting its vital peptidases
Source: Parasit Vectors. 2020 Aug 20;13:426. doi: 10.1186/s13071-020-04279-9 (PMC7439556; doi:10.1186/s13071-020-04279-9)

**Additional file 5: Figure S4.** Amount of nitric oxide released from NOR-5 shown as the concentration of nitrites in the medium. The release was steady and reproducible. Data are shown as means±standard deviation, n=6


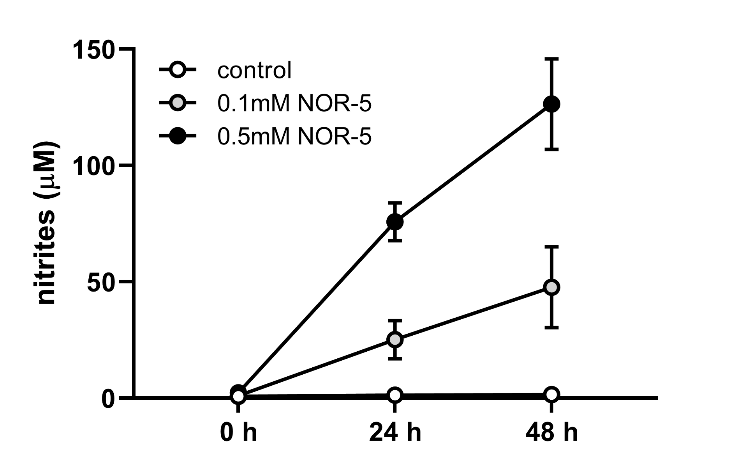

Supplement: Supplementary file 5 — Additional file 5: Figure S4. Amount of nitric oxide released from NOR-5 shown as the concentration of nitrites in the medium. [file 13071_2020_4279_MOESM5_ESM.docx]
