# Supplementary material for: Nitric oxide debilitates the neuropathogenic schistosome Trichobilharzia regenti in mice, partly by inhibiting its vital peptidases
Source: Parasit Vectors. 2020 Aug 20;13:426. doi: 10.1186/s13071-020-04279-9 (PMC7439556; doi:10.1186/s13071-020-04279-9)

**Additional file 6: Figure S5.** Number of body contractions performed by *T. regenti* schistosomula treated *in vitro* by NOR-5, the donor of NO. Thirty-second videos of schistosomula were captured after the 48-hour treatment and analyzed by the ImageJ plugin wrMTrck ([www.phage.dk/plugins/wrmtrck.html](http://www.phage.dk/%20plugins/wrmtrck.html)). Pooled data from four experiment are shown, dots represent data from individual schistosomula. No significant differences were noticed (Kruskal-Wallis test, χ2 = 2.492, df = 2, P = 0.2877)


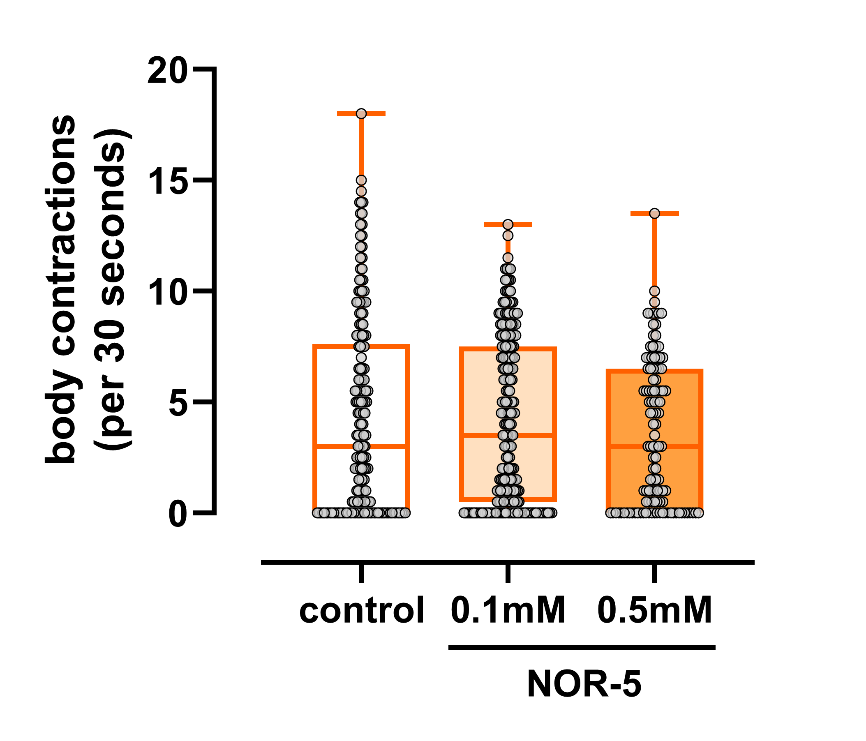

Supplement: Supplementary file 6 — Additional file 6: Figure S5. Number of body contractions performed by T. regenti schistosomula treated in vitro by NOR-5, the donor of NO. [file 13071_2020_4279_MOESM6_ESM.docx]
