# Supplementary material for: Nitric oxide debilitates the neuropathogenic schistosome Trichobilharzia regenti in mice, partly by inhibiting its vital peptidases
Source: Parasit Vectors. 2020 Aug 20;13:426. doi: 10.1186/s13071-020-04279-9 (PMC7439556; doi:10.1186/s13071-020-04279-9)

**Additional file 7: Figure S6.** Ultrastructural changes of *Trichobilharzia regenti* schistosomula treated by 0.5mM NOR-5 visualised by scanning (SEM; a, b) and transmission (TEM; c, d) electron microscopy. Comparison of treated and control schistosomula did not reveal any significant differences in topography (a, b) including size/density of superficial blebs on the tegument (white arrows), or in internal morphology of schistosomula (c, d) including ultrastructure of mitochondria. Abbreviation: T, tegument; SP, spines; BL, basal lamina; CM, circular muscles; LM, longitudinal muscles; M, mitochondria


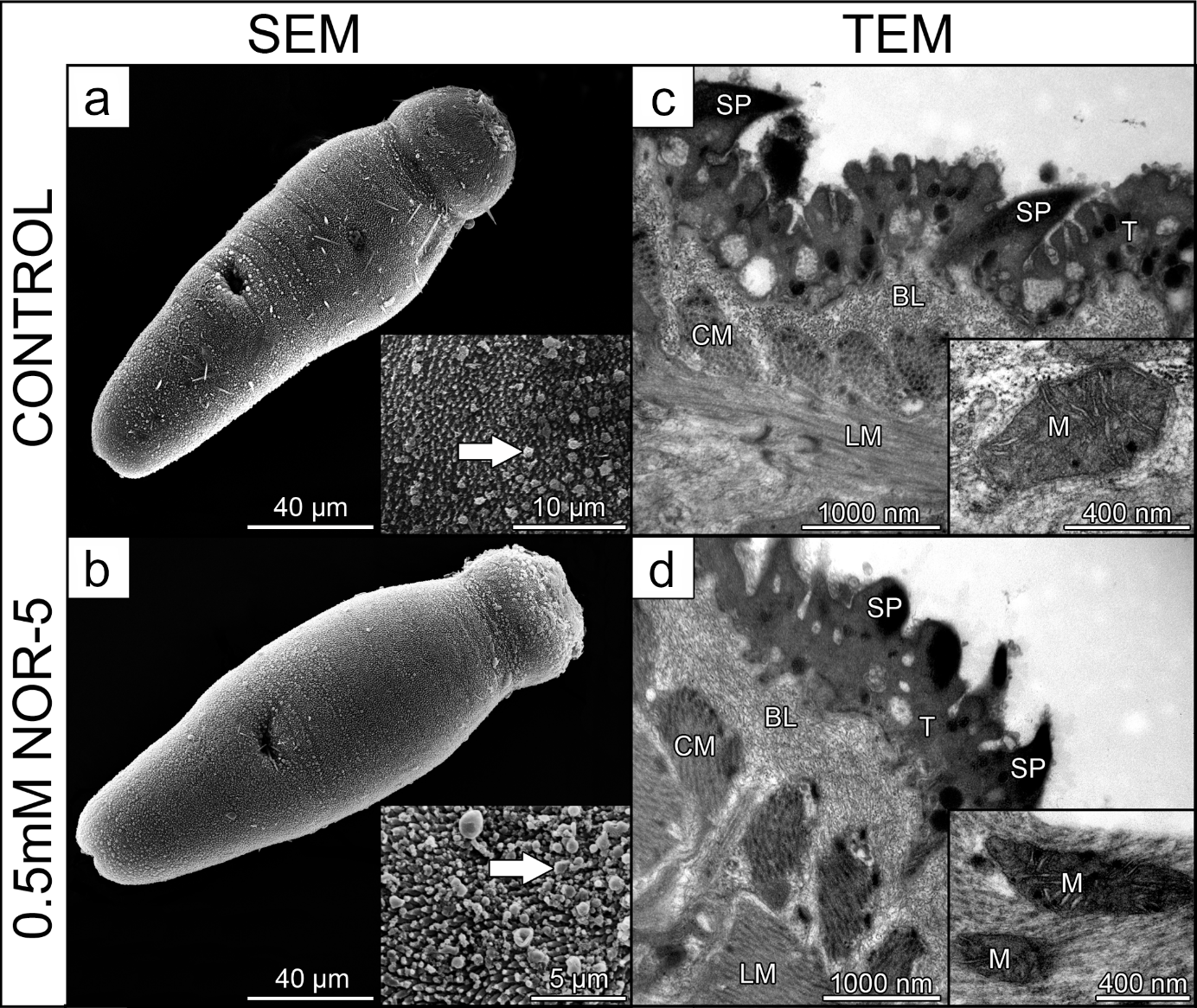

Supplement: Supplementary file 7 — Additional file 7: Figure S6. Ultrastructural changes of Trichobilharzia regenti schistosomula treated by 0.5mM NOR-5 visualised by scanning and transmission electron microscopy. [file 13071_2020_4279_MOESM7_ESM.docx]
